# Atrioventricular Conduction Delay in the Second Trimester Measured by Fetal Magnetocardiography

**DOI:** 10.1155/2014/753953

**Published:** 2014-01-16

**Authors:** Annette Wacker-Gussmann, Henrike Paulsen, Krunoslav Stingl, Johanna Braendle, Rangmar Goelz, Joerg Henes

**Affiliations:** ^1^Department of Neonatology, University Children's Hospital Tuebingen, 72076 Tuebingen, Germany; ^2^fMEG Center, University of Tuebingen, 72076 Tuebingen, Germany; ^3^Department of Obstetrics and Gynecology, University Hospital, 72076 Tuebingen, Germany; ^4^Centre for Interdisciplinary Clinical Immunology, Rheumatology and Auto-Inflammatory Diseases and Department of Internal Medicine II (Oncology, Hematology, Immunology, Rheumatology, Pulmonology), University Hospital, 72076 Tuebingen, Germany

## Abstract

*Introduction*. Fetal AV block in SSA/Ro pregnancies is generally not seen before 18-week gestation and onset is rare after 28-week gestation. If complete AV block appears, it is believed to be irreversible. The purpose of the study was to evaluate precise electrophysiological AV conduction from 18-week gestation onwards. *Patients and Methods*. 21 fetuses of pregnant women with collagen vascular diseases were included in the study group and 59 healthy fetuses served as controls. In addition to fetal echocardiography, fetal magnetocardiography (fMCG) was used to investigate precise electrophysiological fetal cardiac time intervals (fCTIs). *Results*. The PR segment (isoelectric segment between the end of the P wave and the start of the QRS complex) was significantly prolonged (*P* < 0.036 2nd trimester, *P* < 0.023 3rd trimester) in both trimesters within the study group. In fetuses less than 23-week gestational age, a nearly complete separation was found, where a PR segment of 60 ms or greater completely excluded control fetuses. All other fCTIs did not differ significantly. None of the fetuses progressed to a more advanced heart block. *Conclusion*. Slight antibody effects in pregnancy, leading to PR segment prolongation, can already be seen from 18-week gestation onwards by fMCG. Serial fetal Doppler echocardiography and additional fMCG can be useful methods to measure early and precise AV conduction time, to achieve best surveillance for these high-risk pregnancies.

## 1. Introduction

Substantial morbidity and mortality of fetuses in patients with anti-SSA/Ro antibodies in pregnancy are associated with the development of congenital heart block [1–4]. Fetal AV block in SSA/Ro pregnancies is generally not seen before 18-week gestation and onset is rare after 28-week gestation [5]. If complete congenital heart block in these fetuses occurs, it is believed to be irreversible. Nevertheless, intrauterine therapy might be possible, although it is empiric at the moment. The rationale for treatment strategies is to identify the heart block as early as possible and to diminish the inflammatory insult to the heart by lowering the maternal antibodies [6]. Immune-mediated AV block may benefit from in utero treatment with fluorinated steroids, IVIG, or both. Dexamethasone is believed to reduce inflammation [7–9]. Although no clear consensus exists, most clinicians use dexamethasone 4–8 mg/day to treat not only second-degree AV block and recent onset AV block but also severe cardiac dysfunction and hydrops.

Several investigators have reported a transient prolongation of AV conduction time by echocardiography during midtrimester, which was still present on postnatal electrocardiograms (ECG) in 50% of the subjects. The long-term prognosis in these studies was reported as being excellent [10, 11]. These findings might indicate a time frame, where reversal of incomplete block without treatment can be seen.

The methods used are mainly Doppler techniques which measure the mechanical rather than the electrophysiological events to obtain AV intervals. Fetal magnetocardiography (fMCG) might fill in this gap. This innovative method is more precise in detecting fetal conduction and arrhythmias [12–14]. However, fMCG generally captures each of the cardiac time intervals (P wave, QRS complex, and T waves, RR-, PR-, and QT intervals) in fetuses over 24-week gestation; in fetuses below 24-week gestation, which is the most important time frame in the development of AV conduction delay, only QRS and RR intervals can be reliably measured in most fetuses [15, 16]. In consequence, a new analyzing method was investigated. We have previously reported PR segment prolongation, relative to controls, in sixteen 3rd trimester fetuses [17], however, analysis of fMCG intervals at younger gestations was difficult until a new method of signal extraction based on a combination of orthogonal projection and independent component analysis was developed. With the help of this new method, measurements of fCTIs were possible from 18-week gestation onwards [17]. The aim of this study was to investigate precise electrophysiological fetal cardiac time intervals in these high-risk fetuses from 18-week gestation onwards by fMCG. This might help to understand the pathophysiology of reverse AV prolongation.

## 2. Patients and Methods

### 2.1. Patient Population

Baseline characteristics of all 80 patients for this observational study were evaluated with regard to medical history, previous pregnancy outcomes, and medication intake.

21 fetuses of pregnant women with collagen vascular diseases such as systemic lupus erythematosus or Sjogren's syndrome were included in the study group.

At study entry, all patients of the study group fulfilled the following inclusion criteria: presence of anti-SSA/Ro and/or anti-SSB/La antibodies tested by an enzyme linked immunosorbent assay (ELISA) and/or an immunofluorescence test, an immunodiffusion test, and dot blots by a commercial laboratory. Rheumatologic disease was diagnosed by a rheumatologist according to defined criteria [18, 19]. There was no limit concerning the duration of medication intake. Pregnancies over 18-week of gestation with a normal heart beat and a structural normal heart were included.

Exclusion criteria for all neonates were chromosomal abnormalities, malformations, and congenital infections.

Data of the study group were compared to already established norm values of healthy women with uncomplicated pregnancies and normally developing fetuses. Neonatal outcomes defined as normal AV conduction by physical exam and established normative cardiac time intervals for age were assessed by a paediatrician by clinical routine examination, fetal heart rate monitoring, and neonatal ECG.

The study was approved by the ethics review board of the University Hospital Tuebingen. Informed written consent was obtained from each subject.

### 2.2. Methods

At the beginning of the study, conventional echocardiography was performed in the study group to evaluate structural cardiac abnormalities, myocardial function, and fetal heart rate, in addition to regular ultrasound examinations.

fMCG measurements were performed in the study group and in the control group.

Prior to the beginning of each fMCG measurement, ultrasound was performed in all patients to check the fetal position and to localize the fetal heart. Furthermore, cardiotocography was performed over a 20-minute period to obtain complete information about the health of the fetus.

#### 2.2.1. Data Acquisition

The fMCG recordings were acquired using a 156-channel biomagnetic system with channels arranged in a curved array that matched the shape of the gravid abdomen (SARA system, VSM Med Tech Ltd., Port Coquitlam, Canada). All of the measurements were recorded with a sampling rate of 1220.7 Hz in a magnetically shielded room (Vakuumschmelze, Hanau, Germany). The length of the recordings ranged from nine to 35 minutes. Data collection was performed between 18 and 38 weeks gestation, focusing primarily on second trimester.

#### 2.2.2. Data Processing

The analysis of the fetal heart signals was performed according to our previously published work [17]. An automated algorithm using orthogonal projection and independent component analysis was applied to reconstruct the fetal heart signal [20–23]. fCTI evaluation was performed using a custom-made MATLAB program (R2008b, Mathworks, Natick, MA, USA).

The time points identified were used to calculate the duration of the CTI as follows.

P  wave = P_end_ − P_onset_, QRS  complex = QRS_end_ − QRS_onset_. The QT interval was defined as T_end_ − QRS_onset_. The PR interval was determined as P wave + PR segment, whereas PR segment was defined as P_end_ to QRS_onset_. The PR segment may more accurately reflect the AV conduction as the PR interval, because it eliminates any intra-atrial conduction delay reflected by the P wave duration measurement. All fMCG recordings were reviewed by at least one physician who has extended experience in pediatric cardiology.

### 2.3. Statistical Analysis

Statistical analysis was performed using SPSS 20.0 (IBM) for Windows. Normal distribution was tested using Kolmogorov-Smirnov Test. ANCOVA was used for data analysis and the influence of age, gender, and birth weight was tested. *P* < 0.05 was regarded as statistically significant.

## 3. Results

### 3.1. Patient Population

#### 3.1.1. Study Population

21 mothers were included in the study group. The median age of the mothers with systemic lupus erythematosus (*n* = 15 patients) or Sjogren's syndrome (*n* = 6 patients) was 31 years (range 21–46 years) (Table 1). The maternal suppressive therapies in these 21 patients were low-dose prednisolone (*n* = 13), high-dose prednisolone (*n* = 1), hydroxychloroquine (*n* = 12), cyclosporine (*n* = 1), and azathioprine (*n* = 6). Most of the patients received more than one medication.

21 fetuses were measured with a median gestational age of 28 weeks (range 18–38 weeks). Six neonates were too small for gestational age.

#### 3.1.2. Control Population

59 pregnant women were included in the control group. Mean age of the women was 33 years (range 25–50 years). All women were healthy except for one with gestational diabetes. Chronic diseases were found in the following patients: thalassaemia minor (two patients), Crohn's disease (one patient), and factor V Leiden mutation (one patient). Twelve women had a previous history of hypothyroidism but were euthyroid at time of measurement.

59 fetuses were measured with a median gestational age of 32 weeks (range 19–38 weeks). Neonatal outcome revealed 59 healthy newborns. Four fetuses were born prematurely (>32 and <37 weeks of gestation). One newborn was small for gestational age whereas three newborns were large for gestational age.

### 3.2. Fetal Cardiac Time Intervals

Altogether 36 measurements in 21 patients were included in the study group and 63 measurements in 59 subjects were included in the control group. The measurements included measurements >24 weeks gestation of the previous study.

The fCTIs for all patients are shown in Tables 2 and 3.  Table 2 focuses on second trimester (18–26 weeks of gestation), whereas Table 3 has its impact on late gestational ages (27–38 weeks of gestation). As the mean birth weight (shown in Table 1) was significantly smaller (*P* = 0.013) in the study group compared to the control group, *P* values were adjusted for this parameter. In addition, *P* values were adjusted for age and gender.

The PR segment (isoelectric segment between the end of the P wave and the start of the QRS complex) was significantly prolonged in both trimesters within the study group (*P* < 0.036 2nd trimester, *P* < 0.023 3rd trimester). All other CTIs did not differ significantly. Available postnatal ECGs in the study group did not show first-, second- or third-degree AV block.

## 4. Discussion

**Figure 1:**
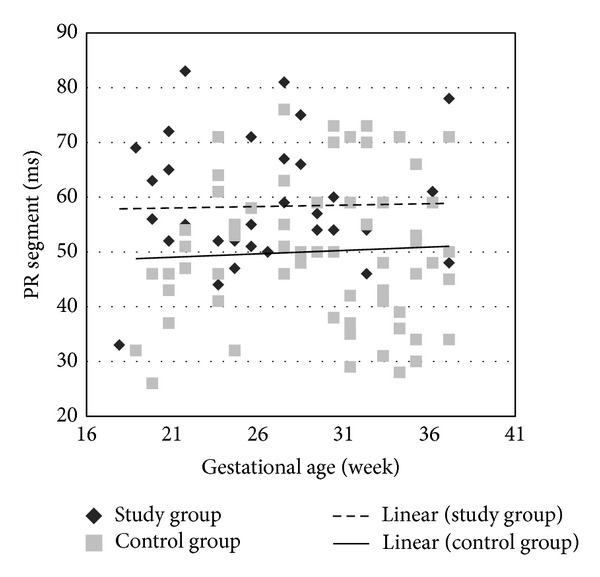
PR segments of study group and control group. Lines indicate 50th %ile measurements.

The main finding in this study was that the PR segment (PR  interval − P  wave), measured by fMCG, was significantly prolonged in the study group not only in the third but also in the second trimester when compared to controls. Four of the study subjects and only one of the control subjects had PR segment measurements equal to or exceeding 75 ms. In fetuses less than 23-week gestational age, a nearly complete separation was found, where a PR segment of 60 ms or greater completely excluded control fetuses (see Figure 1). All other fCTIs did not differ significantly and none of the fetuses progressed to second- or third-degree heart block.

These findings support the concept that mild AV conduction delays may not progress. In a large multicenter study, first-degree AV block did not predict development of advanced AV block, whereas tricuspid regurgitation of moderate or severe degree, and endocardial fibroelastosis, was associated with subsequent onset of 2nd or 3rd degree AV block.

Van Leeuwen and colleagues and Stinstra and colleagues, both, have reported dependency of fCTIs (also the PR segment duration) on gestational age and gender [16, 24, 25]. Additionally, subjects in the study group had a lower weight at birth than those of the control group [26]. However, significant PR segment prolongation was found in the study group, relative to the control group, even after adjusting for all these possible confounding factors.

The new analyzing method improved signal detection and reconstruction in early gestational ages. In consequence, a total of 99 measurements (in 80 patients) could be achieved, 18 under 24-week gestational age. In this study, PR segment prolongation was additionally found in the second trimester (from 18-week gestation onwards) by fMCG. None of the fetuses developed AV block. The prolongation of the PR segment duration might indicate antibody effects already present in the second trimester of pregnancy.

Jaeggi and colleagues reported similar findings by echocardiography from 15 untreated fetuses either with AV prolongation between 2 and 6 *z*-scores or with type one second-degree block. None of the fetuses developed progressive heart block [27].

In addition Sonesson and colleagues reported eight of 24 fetuses who had signs of first-degree block in their study. These AV blocks, measured by Doppler echocardiography, mainly reverted spontaneously. One of these fetuses had progression to complete block, another showed recovery from second-degree to first-degree block with treatment [28]. The lack of progression of first-degree AV block to more severe block makes treatment on the basis of first-degree AV block unnecessary, except perhaps in the most severe prolongation. Rein and colleagues have reported treating patients with dexamethasone for first-degree AV block and attributed the lack of progression as a sign that steroids were effective [29].

Mechanical measurements by Doppler echocardiography and electrophysiological measurements by fMCG, both, show AV prolongation. Stable but also progressive AV prolongation was described in different studies. Therefore serial fetal Doppler echocardiography and additional fMCG measurements of AV time intervals are proposed as a useful method to measure early and precise AV conduction time to achieve best surveillance of these high-risk pregnancies.

In summary, antibody in pregnancy patients might have an effect on the fetal atrioventricular conduction already from early second trimester onwards. But this effect can be reversible and does not consecutively lead to congenital heart block.

To develop new management strategies, fMCG measurements in addition to Doppler echocardiography might be helpful. However, a higher number of these high-risk patients in multicenter studies of this rare condition are probably necessary.

## Figures and Tables

**Table 1:** Baseline characteristics of the mothers, fetuses, and newborns.

	Study group (*n* = 21 patients)	Control group (*n* = 59 patients)
Mother		
Median age of mothers (years; range)	31 (21–46)	33 (25–50)
Prednisolone ≤10 mg (*n*)	13	1
Prednisolone >10 mg (*n*)	1	0
Hydroxychloroquine (*n*)	12	0
Azathioprine (*n*)	6	0
Cyclosporine (*n*)	1	0
Thyroid medication (*n*)	3	12
Aspirin (*n*)	9	1
Fetus		
Median age of fetuses measured in 2nd trimester (weeks; range)	23 (18–26)	24 (19–26)
Median age of fetuses measured in 3rd trimester (weeks; range)	30 (27–38)	33 (28–38)
Newborn		
Male newborns (*n*)	9	34
Female newborns (*n*)	12	25
Preterm >32 and <37 weeks of gestation (*n*)	6	4
Mean birth weight (g; SD)	2845 ± 530	3470 ± 480
Mean birth length (cm; SD)	49 ± 3	51 ± 2
Small for gestational age (*n*)	6	1

**Table 2:** Cardiac time intervals of the study group compared to those of the control group at 18–26 weeks of gestation (*P* value is adjusted for gestational age, gender, and weight).

Characteristics	Study group *n* = 18 measurements mean ± SD (ms)	Control group *n* = 19 measurements mean ± SD (ms)	Statistical significance (ANCOVA)
P wave	38 ± 7	43 ± 6	ns
PR segment	58 ± 11	48 ± 12	*P* = 0.036
PR interval	95 ± 14	91 ± 13	ns
QRS complex	51 ± 12	53 ± 7	ns
T wave	133 ± 37	157 ± 46^#^	ns
QT interval	240 ± 40	259 ± 40^#^	ns

^#^ *n* = 16 measurements due to low identification rate.

**Table 3:** Cardiac time intervals of the study group compared to those of the control group at 27–38 weeks of gestation (*P* value is adjusted for gestational age, gender, and weight).

Characteristics	Study group *n* = 18 measurements mean ± SD (ms)	Control group *n* = 44 measurements mean ± SD (ms)	Statistical significance (ANCOVA)
P wave	48 ± 10	55 ± 13	ns
PR segment	58 ± 11	51 ± 13	*P* = 0.023
PR interval	106 ± 15	104 ± 20	ns
QRS complex	56 ± 5	57 ± 7	ns
T wave	121 ± 13	128 ± 31^#^	ns
QT interval	250 ± 36	250 ± 42^#^	ns

^#^ *n* = 38 measurements due to low identification rate.
